# Role of Viral Molecular Panels in Diagnosing the Etiology of Fever in Infants Younger Than 3 Months

**DOI:** 10.1177/0009922819884582

**Published:** 2019-11-09

**Authors:** Cristina Epalza, Marie Hallin, Laurent Busson, Sara Debulpaep, Paulette De Backer, Olivier Vandenberg, Jack Levy

**Affiliations:** 1 Saint Pierre University Hospital, Bruxelles, Belgium; 2 Université Libre de Bruxelles, Bruxelles, Belgium

**Keywords:** fever under 3 months, etiology, respiratory virus

## Abstract

As infants with proven viral infection present lower risk of bacterial infection, we evaluated how molecular methods detecting viruses on respiratory secretions could contribute to etiological diagnostic of these febrile episodes. From November 2010 to May 2011, we enrolled all febrile infants <90 days presenting to emergency room. Standard workup included viral rapid antigenic test and viral culture on nasopharyngeal aspirate. Samples negative by rapid testing were tested by molecular methods. From 208 febrile episodes (198 infants) with standard techniques, rate of documented microbiological etiology was 13% at emergency department, 47% during hospitalization, and 64% with viral cultures. Molecular methods increased microbiologically documented etiology rate by 12%, to 76%. Contribution of molecular methods was the highest in infants without clinical source of infection, increasing documentation by 18%, from 50% to 68%. Making viral molecular results rapidly available could help identifying a higher proportion of infants at low risk of serious bacterial infection.

## Introduction

The management of febrile infants younger than 3 months in the emergency room (ER) is challenging as they have a higher risk of serious bacterial infection (SBI) than older children and because clinical evaluation has a low sensitivity and specificity in identifying those infants with SBI.^1-6^ Therefore, additional examinations are usually performed to diagnose SBI, or to identify those infants at higher and lower risk of SBI. Accordingly, a large proportion of these infants considered at higher risk of SBI are admitted to the hospital and treated empirically with intravenous antibiotics while awaiting microbiological confirmation, whereas those at lower risk of SBI can be managed as outpatients, provided adequate surveillance can be ascertained.^1-6^

Traditional viral and bacterial diagnostic techniques allow to find the etiology of febrile episodes in about half of the cases only.^7,8^ With the development of molecular techniques, the ability to diagnose viral infections has improved substantially in recent years. Multiple real-time polymerase chain reactions (PCRs) have been developed, increasing the detection rate for cultivable viruses or allowing the detection of non- or difficult-to-cultivate viruses.^9^ More recently, techniques such as multiplex PCRs or microarrays allow the codetection of large panels of viruses in a single assay.^10^ Furthermore, these new tools can now generate accurate results within a few hours.^11^ In several clinical situations, rapid report of microbial pathogens identification from clinical specimens has been shown to significantly improve the management and the outcome of infected patients, enabling rapid adjustment of antibiotic treatments, shortened hospital stay, and lower hospitalization costs.^12,13^ Regarding the management of febrile infants younger than 3 months, several authors have shown that infants presenting a proven viral infection have a significantly lower risk of SBI.^14-18^ An early diagnosis of a viral infection could help consider a larger proportion of febrile infants at lower risk for SBI and lead to a more conservative approach regarding additional invasive procedures, antibiotic treatments, or hospital admission. While the molecular techniques allowing this early diagnosis are more expensive than conventional methods, their real clinical benefits remain poorly studied.

The aims of this prospective study were to evaluate the analytical performances of a multiplex diagnostic tool detecting the most frequent respiratory viruses as compared with our set of homemade real-time PCRs and the potential contribution of these molecular methods to the etiologic diagnosis of febrile episodes in infants younger than 3 months of life.

## Material and Methods

### Study Population

The study was conducted at Saint-Pierre Hospital, a university-affiliated hospital located in downtown Brussels. About 24 000 patients per year attend its pediatric ER. All infants ≤90 days of age admitted to the pediatric ER from November 15, 2010, to May 5, 2011, reporting or presenting a rectal temperature ≥38.0°C, were eligible for this study.

According to published guidelines,^1-6^ our standard procedure for these patients includes a thorough clinical interview, a complete physical examination, and the following laboratory tests: complete blood count, blood bacterial culture, viral rapid antigen testing and viral culture of nasopharyngeal aspirates (NPA), urinalysis, and bacterial culture. Cerebrospinal fluid is obtained for cytology, bacterial culture, and enterovirus (EV) detection by PCR in all infants <28 days or in those 28 to 90 days with toxic aspect or the following laboratory findings: white blood cell ≥15 000/mm^3^ or ≤5000/mm^3^ or C-reactive protein ≥20 mg/L or white cell count >35/µL in urine sediment collected by catheterization. Additional tests such as chest X-ray, stool culture, and stool viral antigen tests are performed whenever clinically indicated. For all patients, an admission is proposed; intravenous empirical antibiotic is started in nearly all infants <28 days and in those older than 28 days with clinical or laboratory alterations.

### NPA for Viral Analysis

Samples received in the laboratory during business hours were processed on receipt. Sample arriving outside business hours were stored at 4°C and processed on the next working day. Standard viral diagnostic procedures consisted in viral culture on confluent Vero, MRC5, and LLC-MK_2_ cell lines (Vircell, Santa-Fé, Spain), and a combination of 3 of the following rapid detection tests according to the season: lateral flow chromatography for influenza A and B (BinaxNOW Influenza A/B, Alere Inc, Waltham, MA), respiratory syncytial virus (RSV; BinaxNOW RSV, Alere Inc) and adenovirus (ADV; Adeno Respi-Strip, Coris BioConcept, Gembloux, Belgium), and direct fluorescent immunoassays for human metapneumovirus (hMPV) and parainfluenza virus (PIV; Argene, Biomérieux, Marcy l’Etoile, France).

If rapid tests were negative, 2 sets of molecular assays were performed: (1) the CLART Pneumovir DNA array (Genomica, Coslada, Spain) detecting influenza A, B, and C; PIV 1, 2, 3, and 4; RSV A and B; hMPV A and B; ADV; EV (solely echoviruses); rhinoviruses; coronavirus 229E; and bocaviruses and (2) a homemade real-time PCRs detecting influenza A and B; PIV 1, 2, 3, and 4; RSV A and B; hMPV A and B; ADV; EV; rhinoviruses; coronavirus 229E, NL63, and OC43; and bocaviruses. Extraction of nucleic acids was arried out with the MagNA Pure LC extraction system (Roche Diagnostics) using the Total Nucleic Acid Large Volume Isolation kit (input volume 800 µL, output volume 200 µL). The Pneumovir assay was performed according to the manufacturer’s instructions; detection and interpretation of the results were conducted by a CARreader (Genomica). All these molecular techniques were performed once a week, except for the PCR targeting EV, which was performed twice a week.

### Establishment of a “Composite” Reference Standard

For NPA with negative rapid tests, as the molecular tests used were presumably more sensitive than the reference standard (viral culture), we constructed, as recommended,^19^ a “composite” reference standard in order to avoid bias in establishing the specificity of the evaluated tests. This “composite” reference standard was constructed as follows: (1) samples were considered as positive for a viral pathogen if they tested positive by at least 2 of the 3 assays used (viral culture, Pneumovir, homemade PCRs) and (2) they were considered negative if they tested negative by at least 2 of the 3 assays. The samples not fitting one of these categories were classified as undetermined. As a consequence, the analytical performances of our PCR detecting coronavirus NL63 and OC43 could not be evaluated, as it was the only method used here that was able to detect these viruses.

### Clinical Data Collection and Analysis

For each patient, the following demographic, clinical, biological, and microbiological data were recorded: age, gender, duration of gestation, immunization status, duration and maximal documented temperature at home and in the ER, symptoms, laboratory results, treatment administered and duration, destination after discharge from ER, complications, and length of stay if hospitalized.

All patients’ files were reviewed by an infectious diseases senior pediatrician, and infants were classified into 4 groups according to clinical symptoms at presentation: (1) respiratory infection, (2) gastrointestinal infection, (3) other focal infection, and (4) fever without source (all infants not included in groups 1, 2, or 3). For each patient, the contribution of the microbiological documentation to the diagnosis was assessed at 4 different time points: (1) at ER discharge, (2) at the end of hospitalization (with all rapid viral tests and bacterial cultures results), (3) when viral culture results became available, and (4) with molecular methods results, according to the composite reference standard definition described above. At each of these 4 steps, patients were classified into 3 diagnostic groups, according to clinical, biological, and microbiological data: (A) proven bacterial or viral etiology, (B) supposed bacterial or viral etiology, and (C) no etiologic diagnosis, according to the definitions listed below.

### Definitions

#### Febrile Episode

Febrile episode was defined as a rectal temperature ≥38.0°C reported or measured at the ER.

#### Serious Bacterial Infection

Serious bacterial infection was defined according to the classical definition of Baker et al.^7^

#### Proven Viral Infection

Proven viral infection was defined as a positive antigen in stool, a positive EV PCR in cerebrospinal fluid, a positive rapid test in NPA, or a positive viral culture and/or a positive molecular test result in NPA (according to the composite reference-standard definition).

#### Suspected Infection

Viral or bacterial suspected infection was defined as clinical signs and nonspecific laboratory tests matching either with viral or bacterial infections but with no microbiological documentation.

#### No Etiology

No etiology was defined as the febrile episodes not matching with any of the definitions described above. If a febrile episode matched with 2 diagnostic definitions, the most severe one was considered as the responsible of the fever.

### Statistical Analysis

Data were recorded on Excel files (Microsoft Office, Windows) and analyzed using descriptive statistics.

### Ethics Committee Approval

Verbal informed consent was obtained from all parents or legal guardians at inclusion. The study was approved by the local ethics committee.

## Results

### Patients and Samples

During the study period, 198 infants were enrolled on admittance to the ER for a total of 208 febrile episodes: 10 infants presented 2 episodes (with a median of 27.5 days between episodes [6-41 days]). Median age was 52 days (6-85 days), 56.3% were males, and 6.7% were prematurely born babies. Median measured for fever in the ER was 37.8°C and 38.5°C at home, for a median duration of 12 hours (0-240 hours) before arrival. For the 42.9% of infants older than 60 days, the immunization program was not yet started (Belgian immunization program is free and starts at 8 weeks of age).

Blood culture, urine culture, and NPA were performed in 96.2%, 94.2%, and 97.6% of patients, respectively. Lumbar puncture was performed in 32.2% of the infants (92.6% of infants <28 days and 56% of older infants with toxic aspect or laboratory alterations), stool viral antigenic tests in 25%, stool culture in 22.6%, and skin pus culture in 0.5% (1 patient). In 200 of the 208 episodes, an NPA was available for complete viral evaluation (3 patients were not sampled, 2 samples were not analyzed with rapid test and viral culture, and 3 others were not analyzed with molecular techniques due to technical issues).

### Analytical Performances of Diagnostic Tools in NPA

**Figure 1.**
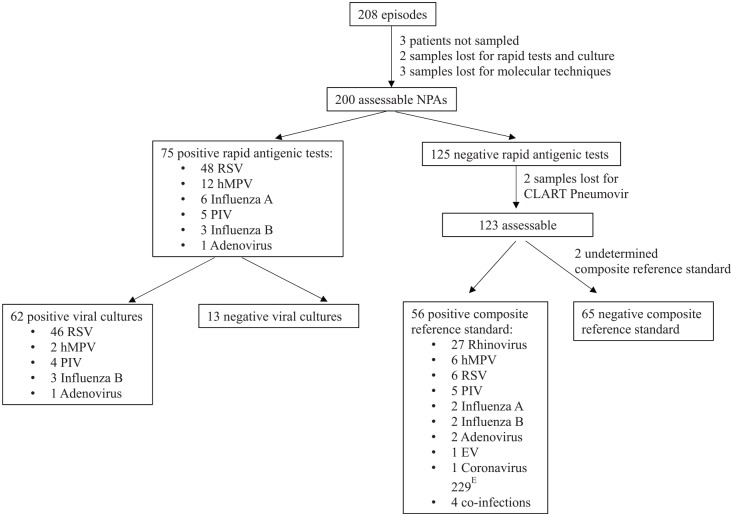
Results of viral diagnostic tests performed on 200 nasopharyngeal aspirates obtained during 208 febrile episodes.

Seventy-five out of 200 NPA (37.5%) had a positive rapid antigen detection test (Figure 1); 62 positive results (82.7%) were confirmed by culture. Among the 13 culture-negative rapid test–positive samples, 10 were hMPV, whose culture growth is known to be difficult. The remaining 125 cases were explored by molecular methods but, as previously said, 2 were not analyzed with Pneumovir assay. From the 123 samples evaluable for analytical performances, the composite reference standard was negative for 65 samples, positive for 56 samples, and undetermined for 2 samples (culture was negative and molecular tests were discordant). Of note, among the 65 “negative” samples, 10 coronaviruses OC43 and 2 coronaviruses NL63 were detected by PCR.

As shown in Table 1, culture was, as expected, the technique with the lowest sensitivity (71%), missing mainly hMPVs, rhinoviruses, and mixed infections; its specificity was 100%. The in-house real-time PCRs showed good specificity (91%) and positive predictive value (88%), but its sensitivity did not reach 80%. The Pneumovir assay, on the other hand, achieved the best sensitivity (96%) and negative predictive value (95%) but lacked specificity (58%), mainly due to 23 results considered as false positives for rhinoviruses according to the composite standard.

**Table 1.** Analytical Performances of Viral Culture, Real-Time PCRs, and CLART Pneumovir Compared With Composite Reference Standard for 121 Evaluable Nasopharyngeal Aspirates.

Diagnostic Technique	Sensitivity (%)	Specificity (%)	Positive Predictive Value (%)	Negative Predictive Value (%)	Overall Concordance (%)	Major Causes of Discrepancies
Viral culture	71	100	100	80	85	Lack of sensitivity for rhinoviruses (n FN = 6), Human metapneumovirus (n FN = 5), coinfection (3/4)
Real-time PCRs	79	91	88	83	83	Lack of sensitivity for rhinoviruses (n FN = 9), lack of specificity for rhinoviruses (n FP = 4)
CLART Pneumovir	96	58	67	95	66	Lack of specificity for rhinoviruses (n FP = 23) and RSV (n, FP = 4)
Corrected for rhinoviruses	*96*	*84*	*89*	*95*	*80*	

Abbreviations: PCR, polymerase chain reaction; FN, false negative; FP, false positive; RSV, respiratory syncytial virus.

### Clinical Outcome and Etiologic Diagnosis

**Figure 2.**
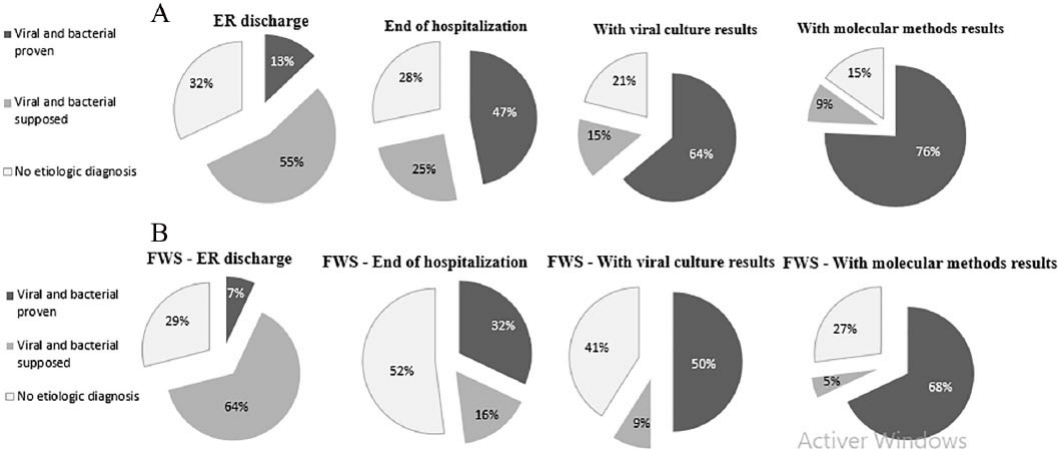
Rate of microbiologically documented diagnosis at various time points. (A) In the general group. (B) In the group of infants with fever without source.

Eighty-four percent of episodes (n = 175) led to hospitalization and 87 episodes (42%) to intravenous empirical antibiotic treatment. Using our standard protocol, the rate of documented microbiological etiology was 13% at ER discharge, 47% at the end of hospitalization, and 64% when viral cultures results became available. Molecular methods increased the documented etiology rate by 12%, to a total of 76% of all episodes (Figure 2). The highest rate of documented episodes was achieved for infants with respiratory symptoms at presentation (92.6%), followed by those with gastrointestinal symptoms (76.5%). The contribution of molecular methods to establish the etiology of the febrile episode was the highest for infants with fever without clinical focus, increasing the rate of microbiological documentation by 18%, to a total of 68% (Figure 2).

**Figure 3.**
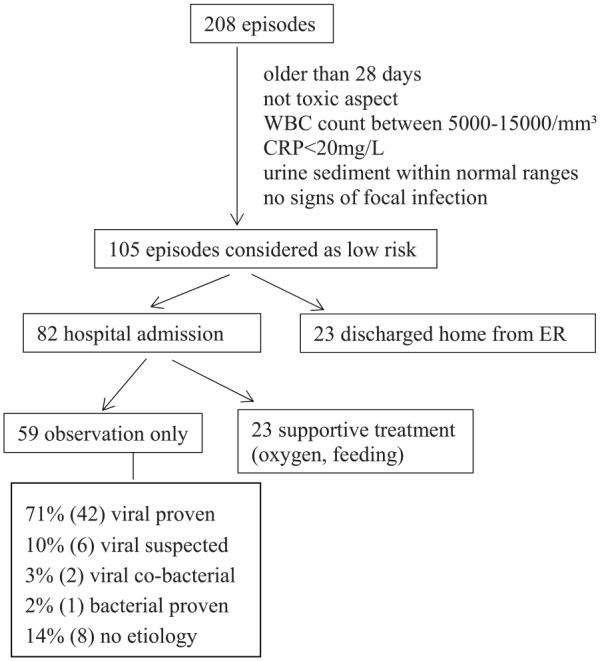
Outcome of febrile infants considered as low risk of serious bacterial infection.

Among the 76% of episodes with documented etiology, SBI was diagnosed in 15 patients (7%): 11 urinary tract infections (UTIs), 3 bacterial enteritis, and 1 finger cellulitis (Table 2). Viruses were detected in the NPA of 5 of these 15 infants (33%): 1 RSV, 1 rhinovirus, 1 PIV, 1 coronavirus NL63, and 1 cytomegalovirus. The remaining 69% were “documented viral infections.” Twenty-one of the 143 episodes (15%) with a proven viral etiology were suspected to have a bacterial coinfection on clinical basis or because they had laboratory signs of inflammation. These 21 episodes occurred all in infants with respiratory symptoms at presentation. In 105 episodes (50.5%), infants were considered as low risk for bacterial infection after workup in the ER (Figure 3); 82 (78%) of these infants were hospitalized, 59 of them (77%) only for clinical observation, while 23 required supportive treatment. One single episode classified as “low risk” at ER discharge ended to be a “bacterial proven infection,” namely, a *Campylobacter jejuni* enteritis. Among the 59 infants with febrile episodes considered at low risk of SBI and hospitalized for observation, 42 (71%) ended to have a documented viral infection.

**Table 2.** Description of the 15 Confirmed Episodes of SBI.

SBI, n = 15 (7%)			Virus Detected in SBI Infants	
Urinary tract infection	11	8 *Escherichia coli*, 2 *Klebsiella pneumoniae*, and 1 *Escherichia coli* and *Klebsiella oxytoca*	3	RSV, PIV, coronavirus NL63
Bacterial gastroenteritis	3	2 *Campylobacter jejuni*, 1 *Salmonella enteritidis*	2	Rhinovirus, cytomegalovirus
Finger cellulitis	1	*Staphylococcus aureus*		

Abbreviations: SBI, serious bacterial infection; RSV, respiratory syncytial virus; PIV, parainfluenza virus.

## Discussion

This is the first prospective study reporting the contribution of a large panel of techniques, including molecular methods for multiple respiratory viruses, to establish the etiology of febrile episodes in infants younger than 3 months. We evaluated the possible contribution on clinical decisions in parallel with the chronology of the availability of the laboratory results that led to episode documentation. Our study presents the limitation of having been conducted mainly during the autumn-winter season in a single center. This can explain the important rate of infants presenting respiratory symptoms. Nevertheless, the rate of SBI is similar to those reported in the literature,^2,3,5^ as is the predominance of UTI among our SBI, demonstrating that our cohort can be considered as representative of the etiologic case mix usually observed among febrile episodes in infants younger than 3 months.

Identifying infants at risk of SBI among those presenting a febrile episode remains an important challenge, and several scales based on clinical, biological, and microbiological data have been recommended.^1-6^ In the past years, important advances have been made in terms of laboratory techniques that either help better identify high-risk infants or confirm SBI more rapidly, such as procalcitonin level^20-24^ or rapid bacterial identification from blood culture by MALDI-TOF (matrix-assisted laser desorption/ionization–time-of-flight mass spectrometry).^25^ Other authors have focused on detecting infants at low risk of SBI using clinical scores.^26^ Documenting a viral infection has also been shown to contribute to identify febrile infants at low risk of SBI.^9-13^ However, as having a proven viral infection does not fully rule out the risk of SBI (especially UTI),^15-17^ viral tests cannot replace blood or urine analysis but must be seen as additional tools in the management of these patients.

Current routine viral tests are rapid and relatively sensitive in pediatric population but are available for a limited number of viruses only. As a consequence, viral infection is not always documented on time to affect the clinical care.

In our study, the infants considered at “low risk of SBI” when discharged from the ER that finally turned out to have a proven viral infection represented about 20% of the total cohort (n = 42). These numbers are similar to those published by Huppler et al, who identified in a large meta-analysis of 21 studies a similar rate of infants (30%) that were observed without receiving empiric antibiotic therapy or sent back home, after being identified at “low-risk.”^27^

This population, namely, “low risk” infants with a presumed viral infection, is probably the population that could benefit the most from adding molecular tools to their management, sparing them the bundle of downsides associated with hospitalization and empiric antibiotic therapy, including costs, adverse effects, development of resistant organisms, nosocomial infections, and psychosocial stress on family dynamics.

The present evaluation demonstrates that molecular techniques greatly improve the detection rate of viral infections, especially in the challenging group of febrile infants without clinical source, among which the increase in microbiological documentation was nearly 20%. The CLART Pneumovir was the most powerful tool tested, multiplexing 11 viral targets with a sensitivity rate of 96% and a negative predictive value of 95%. However, it also presented 2 potential drawbacks: its lack of specificity (false-positive results for rhinoviruses) and the fact it does not target all circulating coronaviruses.

Unfortunately, as the molecular methods used here are expensive and need trained staff, they are not yet routinely used in the management of febrile infants younger than 3 months.^28^ Furthermore, to be useful in clinical practice, the results of these techniques should be available during the timespan in which patient’s management, treatment, and follow-up are decided. New “sample-in, answer-out” point-of-care platforms that enable fully automated detection of comprehensive panels of respiratory pathogens in about 1 hour are now available and should soon enable this expected improvement of patient’s management.^29^

In conclusion, our study demonstrates that the use of molecular techniques increases to 76% the proportion of documented etiology in febrile episodes in infants younger than 3 months. We have also identified the population in which these techniques have the highest contribution. Making these tests available 24 hours/24 and 7 days/7 could help lightening the management of these patients.

Our study provides adequate information to design a prospective study that could fully assess the contribution of new, rapid, point-of-care, multiplex molecular tools to the management of febrile infants younger than 3 months.

## Author Contributions

CE: conceptualized and designed the study, coordinated patient’s recruitment, designed the data collection instruments, coordinated and supervised data collection, drafted the initial manuscript, and approved the final manuscript as submitted. MH: supervised the molecular techniques, carried out the laboratory data analysis, critically reviewed and revised the manuscript, and approved the final manuscript as submitted. LB: supervised the laboratory techniques, reviewed the manuscript, and approved the final manuscript as submitted. SD: helped in patient’s recruitment, reviewed the manuscript, and approved the final manuscript as submitted. PDB: helped in patient’s recruitment, reviewed the manuscript, and approved the final manuscript as submitted. OV: reviewed the manuscript and approved the final manuscript as submitted. JL: conceptualized and designed the study, critically reviewed and revised the manuscript, and approved the final manuscript as submitted. All authors approved the final manuscript as submitted and agree to be accountable for all aspects of the work.

## Supplemental Material

20130121VR3M_resultats_Viro-CDM_MH_GV_final_CE – Supplemental material for Role of Viral Molecular Panels in Diagnosing the Etiology of Fever in Infants Younger Than 3 Months Click here for additional data file. Supplemental material, 20130121VR3M_resultats_Viro-CDM_MH_GV_final_CE for Role of Viral Molecular Panels in Diagnosing the Etiology of Fever in Infants Younger Than 3 Months by Cristina Epalza, Marie Hallin, Laurent Busson, Sara Debulpaep, Paulette De Backer, Olivier Vandenberg and Jack Levy in Clinical Pediatrics
